# Metagenomics and Non‐Targeted Metabolomics Reveal the Role of Gut Microbiota and Its Metabolites in Brain Metastasis of Non‐Small Cell Lung Cancer

**DOI:** 10.1111/1759-7714.70068

**Published:** 2025-04-22

**Authors:** Chen‐Guang Liu, Mei‐Xi Lin, Yu Xin, Man Sun, Jia Cui, Dan Liu, Dan Zang, Jun Chen

**Affiliations:** ^1^ Department of Oncology The Second Hospital of Dalian Medical University Dalian China

**Keywords:** brain metastasis, gut microbiota, metabolomics, metagenomics, non‐small cell lung cancer

## Abstract

**Background:**

Brain metastasis is a common and severe complication in non‐small cell lung cancer (NSCLC) patients, significantly affecting prognosis. However, the role of gut microbiota and its metabolites in NSCLC brain metastasis remains poorly understood. This study aims to explore the relationship between gut microbiota, metabolites, and the development of brain metastasis in NSCLC.

**Methods:**

We conducted an integrative analysis combining metagenomics and non‐targeted metabolomics on baseline fecal samples from NSCLC patients with brain metastasis (*n* = 18) and those without distant metastasis (*n* = 12). Gut microbiota composition and metabolite profiles were detected and analyzed, and statistical methods, including machine learning models, were applied to identify differences and potential biomarkers.

**Results:**

Significant differences in gut microbiota composition were found between the two groups, with higher microbial diversity observed in patients with brain metastasis. Specific genera, such as *Paenibacillus*, *Fournierella*, and *Adlercreutzia*, were enriched in the brain metastasis group. Metabolomic analysis revealed altered levels of short‐chain fatty acids and other metabolites associated with immune modulation and vascular permeability, including angiotensin (1–7). These changes were linked to the metastatic process and may influence brain metastasis development. Furthermore, machine learning models identified key biomarkers, such as *Raoultibacter*, *Mobilibacterium*, and *N*‐acetyl‐L‐glutamic acid, which could serve as valuable indicators for brain metastasis.

**Conclusions:**

Our findings suggest that gut microbiota dysbiosis and its metabolic products may contribute to the development of brain metastasis in NSCLC. The identification of microbiota‐derived biomarkers holds potential for early detection and therapeutic intervention in NSCLC brain metastasis.

## Graphical abstract

This study investigates the role of gut microbiota and its metabolites in NSCLC brain metastasis. Through metagenomic and metabolomic analyzes of baseline fecal samples from NSCLC patients with brain metastasis and those without distant metastasis, we identified significant differences in specific microbial taxa and metabolites. These findings suggest that gut dysbiosis and metabolic alterations mediate brain metastasis, offering potential biomarkers for early diagnosis and prognosis.

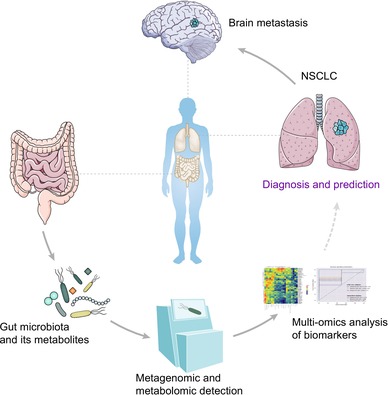

## 1 Introduction

Non‐small cell lung cancer (NSCLC) is the most common type of lung cancer, accounting for approximately 85% of all cases, and remains the leading cause of cancer‐related death worldwide [1, 2]. Despite advancements in diagnostic and treatment strategies, NSCLC often progresses to advanced stages, where it can metastasize to various organs, including the brain. Brain metastasis is a particularly dire complication of NSCLC, occurring in up to 40% of patients with advanced disease [3]. Brain metastases are associated with a significantly poorer prognosis and can lead to severe neurological symptoms, such as headaches, seizures, and cognitive impairments. The pathophysiology underlying the preferential spread of NSCLC to the brain is not yet fully understood, although the blood–brain barrier, tumor microenvironment interactions, and molecular factors such as genetic mutations are thought to play pivotal roles in facilitating this process [4]. Given the devastating effects of brain metastasis, there is an urgent need to explore novel biomarkers and therapeutic approaches to predict and treat brain metastasis in NSCLC patients [5].

The gut microbiota, consisting of trillions of microbes, is increasingly recognized for its essential role in maintaining host health and regulating various physiological processes, including immune function, metabolism, and even neural signaling [6]. The gut microbiota communicates with the immune system and other organs through both direct and indirect pathways, affecting systemic health and disease susceptibility. Dysbiosis, or an imbalance in the gut microbiota, has been linked to numerous health disorders, including gastrointestinal diseases, autoimmune conditions, and cancers [7, 8]. Of particular interest is the influence of gut microbiota on cancer progression. Research has shown that the composition of gut microbial communities can modulate immune responses, affect tumor growth, and influence the response to cancer therapies [9]. This has led to the concept of the “gut‐tumor axis,” which describes the complex interplay between the gut microbiome and cancer progression, including its potential role in metastasis. Evidence is mounting that alterations in the gut microbiota may not only affect primary tumor growth but also modulate the metastatic potential of cancer cells [10, 11].

In the context of NSCLC, changes in gut microbiota composition have been linked to the ability of tumor cells to metastasize to distant organs, including the brain. Gut microbiota dysbiosis is thought to contribute to an altered immune response that facilitates the dissemination of cancer cells via the bloodstream and their subsequent colonization of the brain [12]. Moreover, emerging data suggest that gut microbiota‐derived metabolites can affect the permeability of the blood–brain barrier, providing a potential mechanism by which microbial alterations influence brain metastasis in NSCLC [13, 14].

The relationship between gut microbiota, their metabolites, and cancer metastasis is particularly relevant in the context of NSCLC brain metastasis. Several studies have suggested that the presence of certain microbial species and their metabolites may promote or inhibit the spread of cancer cells to the brain. These findings suggest that gut microbiota and their metabolic products may play a pivotal role in the development of brain metastasis in NSCLC patients. The identification of specific microbial taxa and metabolites that influence brain metastasis could lead to the development of microbiota‐based biomarkers and therapeutic strategies aimed at preventing or treating metastasis in NSCLC.

In this study, we aim to investigate the relationship between gut microbiota and NSCLC brain metastasis through an integrative approach combining metagenomic and non‐targeted metabolomics analyzes. By examining fecal samples from NSCLC patients with brain metastasis and those without distant metastasis, we seek to identify microbial and metabolic signatures associated with brain metastasis and explore the potential mechanisms by which gut microbiota contribute to this process. Our findings may provide novel insights into the gut‐brain axis in NSCLC metastasis and offer new avenues for therapeutic interventions targeting the microbiome to prevent or treat brain metastasis in NSCLC.

## 2 Materials and Methods

### 2.1 Ethics

The study protocol and the informed consent form were approved by the Ethics Review Committee and the Scientific Review Committee of the Second Hospital of Dalian Medical University (Ethics Approval No. 2022‐173). All patients enrolled and assessed in this study provided written informed consent. The study was conducted in accordance with the revised Declaration of Helsinki and relevant guidelines.

### 2.2 Study Subjects and Sample Collection

Fecal baseline samples were collected from NSCLC patients with brain metastasis (*n* = 18) and those without distant metastasis (*n* = 12) between January 2022 and December 2023. Specifically, participants who met the following inclusion criteria were eligible for this study: (1) full understanding of and voluntary participation in the study, with signed informed consent; (2) histologically confirmed diagnosis of primary NSCLC; (3) age between 18 and 80 years (4) ECOG performance status of 0–1; (5) presence of assessable lesions by CT or MRI, with at least one evaluation result. Participants were excluded based on the following exclusion criteria: (1) autoimmune diseases or gastrointestinal disorders; (2) a second primary malignant tumor; (3) use of antibiotics, laxatives, probiotics, or high‐dose corticosteroids within the past 3 months; (4) infectious diseases such as viral hepatitis, HIV, or syphilis.

All participants were instructed on the procedure for fecal collection. Specifically, patients were guided to deposit fresh feces into sterile containers, which were then placed in foam boxes with ice packs. The samples were transported within 30 min to a liquid nitrogen tank for rapid freezing, and subsequently transferred to and stored at −80°C until further metagenomic and non‐targeted metabolomics sequencing analysis.

### 2.3 Metagenomic Sequencing

Total genomic DNA was extracted from fecal samples using the PowerSoil DNA Isolation kit (Mo Bio Laboratories). DNA quality and quantity were assessed using the Qubit dsDNA HS Assay Kit on a Qubit 3.0 Fluorometer (Life Technologies) and 1% agarose gel electrophoresis. Paired‐end libraries (insert size, ~350 bp) were prepared using the VAHTS Universal Plus DNA Library Prep Kit (Vazyme Biotech) and sequenced on an Illumina NovaSeq 6000 platform using 150‐bp paired‐end mode. Raw sequence data were quality‐filtered using Trimmomatic v0.33 to remove adapters and low‐quality reads (quality score < 20, sliding window size of 50 bp, sequence length < 120 bp). Reads were aligned to the human genome using Bowtie2 (version 2.2.4) to remove host contamination. Clean reads were assembled using MEGAHIT, and contigs ≥ 300 bp were selected for further analysis. Assembly quality was assessed using QUAST software version 2.3.

### 2.4 Untargeted Metabolomics Analysis

Untargeted metabolomics analysis was performed using a Waters UPLC Acquity I‐Class PLUS system coupled with a Waters UPLC Xevo G2‐XS QTof mass spectrometer. Metabolites were separated on an Acquity UPLC HSS T3 column (1.8 μm, 2.1 × 100 mm). The mobile phases consisted of 0.1% formic acid in water and 0.1% formic acid in acetonitrile. Mass spectrometry was conducted in both positive and negative ion modes (ESI+ and ESI−). Data were acquired using MSe mode with a low energy of 2 V and high energy ramping from 10 to 40 V. Raw data were processed using Progenesis QI software for peak extraction, alignment, and metabolite identification using the METLIN database.

### 2.5 Statistical Analysis

Statistical analysis of clinical data was performed by GraphPad Prism 9.5 and SPSS 25.0. Descriptive statistics, including mean, standard deviation, and frequency, were used to summarize baseline demographic and clinical characteristics. Statistical significance between two groups was determined using unpaired Student's *t*‐tests. Correlation analyzes were performed using Spearman's correlation coefficient. All statistical tests were two‐sided, and *p*‐value < 0.05 were considered statistically significant.

### 2.6 Bioinformatics Data Analysis

The BMKCloud platform (www.biomarker.com.cn) was used for metagenomics, non‐targeted metabolomics assay analysis, and multi‐omics combination analysis.

#### 2.6.1 Species Diversity Analysis

We assessed alpha diversity using the ACE, Chao1, Shannon, and Simpson indices. Beta diversity was analyzed using the Binary_jaccard algorithm distance matrix, followed by Analysis of similarities (Anosim) and Permutational multivariate analysis of variance (PerMANOVA) using the vegan package in R.

#### 2.6.2 Intergroup Species Differences

The Wilcoxon rank‐sum test was used to evaluate species abundance differences at the genus level between the two groups. Line Discriminant Analysis (LDA) Effect Size (LEfSe) analysis was performed to examine species evolutionary branches and LDA value distributions.

#### 2.6.3 Species Correlation Analysis

We selected the top 80 species by abundance (from phylum to species level) and performed correlation analysis using the Spearman method based on the abundance and variation of species in each sample. We then conducted statistical tests, selecting correlations greater than 0.5 and with a *p*‐value less than 0.05 to build a correlation network.

#### 2.6.4 Metabolite Annotation

We used databases such as the Kyoto Encyclopedia of Genes and Genomes (KEGG) database, Human Metabolome Database (HMDB), and Lipid Metabolites and Pathways Strategy (LIPID MAPS) for the annotation of all identified metabolites.

#### 2.6.5 Intergroup Differential Metabolite Analysis

Orthogonal Partial Least Squares Discriminant Analysis (OPLS‐DA) was used to compare the smajor distribution differences of metabolites between the two groups. Differential metabolites were further selected based on the Variable Importance in Projection (VIP) score from the OPLS‐DA model, along with univariate analysis (*p*‐value and fold change).

#### 2.6.6 KEGG Functional Annotation and Enrichment Analysis of Differential Metabolites

The differential metabolites were annotated using the KEGG database and subjected to enrichment analysis using the hypergeometric test through the clusterProfiler package.

#### 2.6.7 Correlation Analysis Between Differential Metabolites and Species Abundance

After standardizing the abundance data, we used Pearson correlation analysis to examine the relationship between differential metabolites and species abundance. Additionally, we performed multi‐omics integrative analysis using the Omicade4 package in R.

#### 2.6.8 Random Forest Model Construction

In the random forest model, multiple decision trees are built using random subsets of both training samples and features. Each tree is constructed using a bootstrap sampling technique, where a random subset of the training data is selected with replacement. This random sampling process introduces diversity among the individual trees, leading to slightly different prediction errors. The final prediction is made by averaging the predictions of all individual trees, which helps to reduce overfitting and improve the overall model accuracy. The performance of the random forest model was assessed using 10‐fold cross‐validation to ensure robustness and prevent overfitting. The variable importance was calculated based on the Mean Decrease in Accuracy (MDA) and Mean Decrease in Gini (MDG) scores, which allowed for the identification of the most representative microbial species and metabolites.

## 3 Results

### 3.1 Patient Baseline Characteristics

A total of 30 eligible patients were enrolled in this study, with ages ranging from 41 to 79 years old and a mean age of 62.5 years old. Among them, 16 were male and 14 were female. The cohort included 8 patients with lung squamous cell carcinoma and 22 patients with lung adenocarcinoma. In terms of clinical tumor staging, patients without metastasis were distributed in stage I–III, while all patients with brain metastasis were in stage IV. There were no significant differences between the non‐metastasis and brain metastasis groups in terms of demographic characteristics, including age, gender, smoking and alcohol consumption habits, pathological types, genetic mutation profiles, tumor (T) stage, lymph node (N) stage, family history of cancer, or body mass index (BMI). These findings indicated that the baseline characteristics of the two groups were well‐balanced and comparable (Table 1).

**TABLE 1:** Baseline clinical characteristics of study participants.

Characteristic		Non‐metastasis (*n* = 12)	Brain metastasis (*n* = 18)	*p*
Age (years)		63.2 ± 7.4	62.1 ± 10.9	0.760
Sex, *n* (%)	Male	8 (66.7%)	8 (44.4%)	0.232
	Female	4 (33.3%)	10 (55.6%)	
Smoking status, *n* (%)	Current smoker	3 (25.0%)	2 (11.1%)	0.134
	Former smoker	3 (25.0%)	1 (5.6%)	
	Never smoker	6 (50.0%)	15 (83.3%)	
Alcohol consumption, *n* (%)	Current drinker	1 (8.3%)	2 (11.1%)	0.892
	Former drinker	2 (16.7%)	2 (11.1%)	
	Never drinker	9 (75.0%)	14 (77.8%)	
Pathological types	Adenocarcinoma	10 (83.3%)	12 (66.7%)	0.419
	Squamous cell carcinoma	2 (16.7%)	6 (33.3%)	
Genetic mutation profiles	EGFR 21 L858R	2 (20%)	7 (58.4%)	0.141
	EGFR Exon 19 Del	1 (10%)	2 (16.7%)	
	EGFR T790M	1 (10%)	1 (8.3%)	
	ALK	1 (10%)	1 (8.3%)	
	BRAF V600E	1 (10%)	0 (0%)	
	ERBB2	0	1 (8.3%)	
	No genetic mutations	4 (40%)	0 (0%)	
Tumor (*T*) stage	1	5 (41.7%)	3 (16.7%)	0.128
	2	4 (33.3%)	6 (33.3%)	
	3	2 (16.7%)	1 (5.6%)	
	4	1 (8.3%)	8 (44.4%)	
Lymph node (*N*) stage	0	2 (16.7%)	1 (5.6%)	0.583
	1	0 (0%)	2 (11.1%)	
	2	3 (25.0%)	6 (33.3%)	
	3	7 (58.3%)	9 (50%)	
Family history of cancer, *n* (%)	None	12 (100%)	14 (77.8%)	0.215
	Lung cancer	0 (0%)	1 (5.6%)	
	Other cancers	0 (0%)	3 (16.7%)	
BMI (kg/m^2^)		24.93 ± 3.08	23.67 ± 3.31	0.212

*Note:* Data are presented as mean ± SD or *n* (%).

Abbreviations: ALK, anaplastic lymphoma kinase; BMI, body mass index; BRAF V600E, B‐Raf proto‐oncogene serine/threonine kinase valine 600 glutamic acid; EGFR 21 L858R, epidermal growth factor receptor 21 leucine 858 arginine; EGFR Exon 19 Del, epidermal growth factor receptor exon 19 deletion; EGFR T790M, epidermal growth factor receptor threonine 790 methionine; ERBB2, Erb‐B2 receptor tyrosine kinase 2.

### 3.2 Characterization of Gut Microbiota in NSCLC Patients Based on Metagenomic Analysis

**FIGURE 1:**
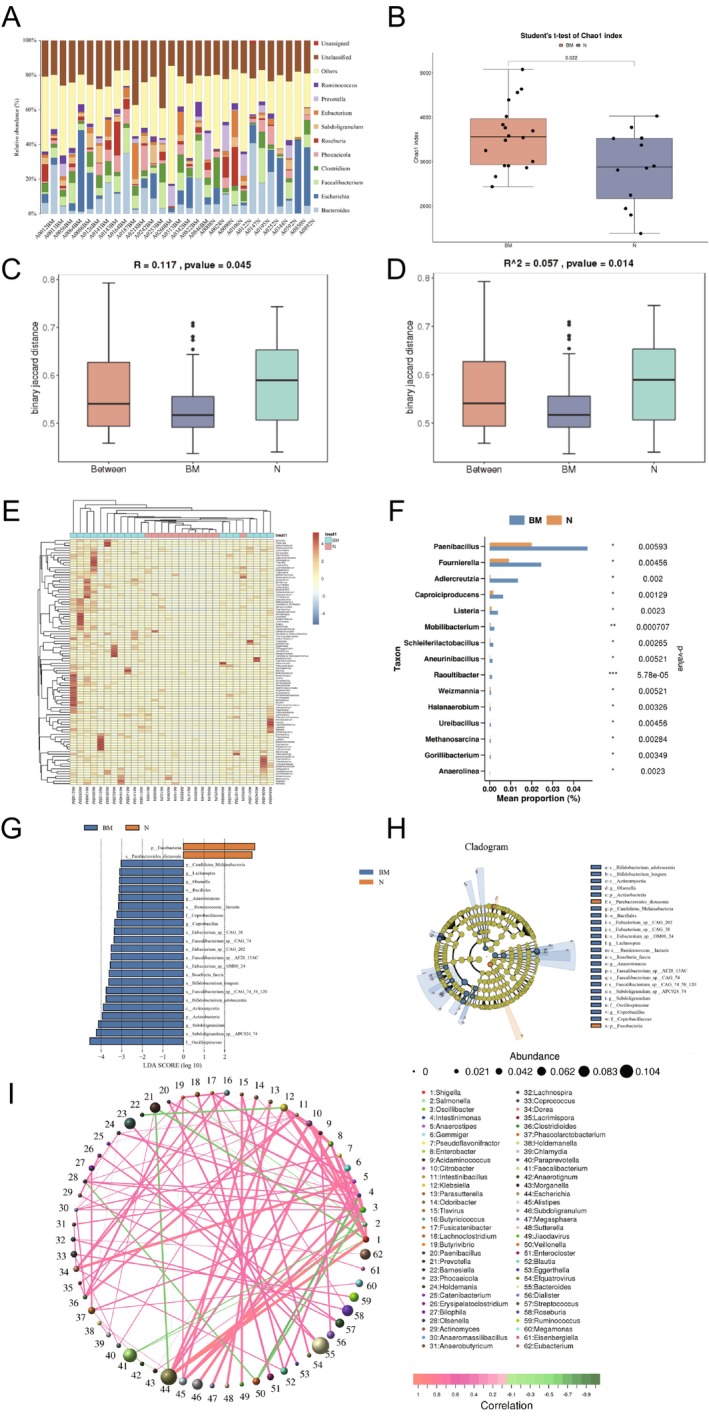
Gut microbiota composition and diversity in NSCLC patients with brain metastasis and without distant metastasis. (A) Genus‐level species composition of fecal samples from all NSCLC patients. (B) The difference in Chao1 index between NSCLC patients without distant metastasis (N) and those with brain metastasis (BM) was based on α‐diversity analysis. Each dot represents an individual patient. (C, D) β‐diversity analysis showing differences in microbial community structure between the two groups. (E, F) Differential species abundance between the two groups. (G) LEfSe analysis showing the distribution of LDA scores between the two groups. (H) Circular cladogram illustrating the differences in microbial community or species structure between groups. (I) Correlation network of microbial species at the genus level.

We conducted metagenomic analysis on baseline fecal samples collected from 18 NSCLC patients with brain metastasis and 12 patients without distant metastasis. The average sequencing depth used to detect gut microbiota and genes was saturated, with a depth of 6G, providing profiles across six kingdoms: Eukaryota, Metazoa, Fungi, Archaea, Viruses, and Bacteria. This included 160 phyla, 149 classes, 314 orders, 676 families, 2447 genera, and 11 976 species (Figure 1A). Alpha diversity analysis based on species showed that patients with brain metastasis had significantly higher ACE and Chao1 indices compared to NSCLC patients without distant metastasis (*p* = 0.022), and there was also a certain difference between Shannon index and Simpson index, although not significant(*p* = 0.082, *p* = 0.27), indicating higher microbial species richness in the gut microbiota of brain metastasis patients, with a notable increase in rare species (Figures 1B and S1). Beta diversity analysis, using the Binary_jaccard algorithm distance matrix, followed by Anosim and PerMANOVA analysis in R's vegan package, demonstrated significant differences in the gut microbiota community structure between the two groups (*p* = 0.045, *p* = 0.014) (Figure 1C,D).

Differences in species abundance between the two groups at the generic level revealed a significant dysbiosis in the gut microbiota of patients with brain metastasis compared to those without distant metastasis, with many microbial abundances showing significant changes in the brain metastasis group (Figure 1E). At the genus level, the abundance of *Paenibacillus*, *Fournierella*, *Adlercreutzia*, *Caproiciproducens*, and *Listeria* was significantly increased in the brain metastasis group (Figure 1F). LEfSe analysis at the species level revealed significant differences in various gut microbiota between the two groups (Figure 1G,H). *Fusobacteria* and *Parabacteroides_distasonis* were upregulated in the non‐metastasis group, whereas *Oscillospiraceae*, *Subdoligranulum*, and *Actinobacteria* were notably upregulated in the brain metastasis group. These genera have been previously identified as opportunistic human pathogens in several studies [15, 16, 17].

To further investigate the significance of gut microbiota dysbiosis in brain metastasis of NSCLC, we performed functional annotation using the KEGG database. We found significant and complex interrelationships between species, suggesting that microbial species may interact with each other. For instance, *Escherichia*, *Shigella*, and *Salmonella* exhibited a significant positive correlation, indicating that gut microbiota dysbiosis may be closely associated with the onset of various diseases. In contrast, the probiotic *Veillonella* showed a significant negative correlation with *Oscillibacter* and *Intestinimonas*. These three genera are involved in the production of short‐chain fatty acids (SCFAs), highlighting the intricate metabolic interactions within the gut microbiota. Such interspecies symbiotic relationships are critical for maintaining the ecological balance and metabolic health of the host's gut microbiome (Figure 1I).

### 3.3 Metabolic Profiling of Gut Microbiota in NSCLC Patients Revealed by Non‐Targeted Metabolomics Analysis

**FIGURE 2:**
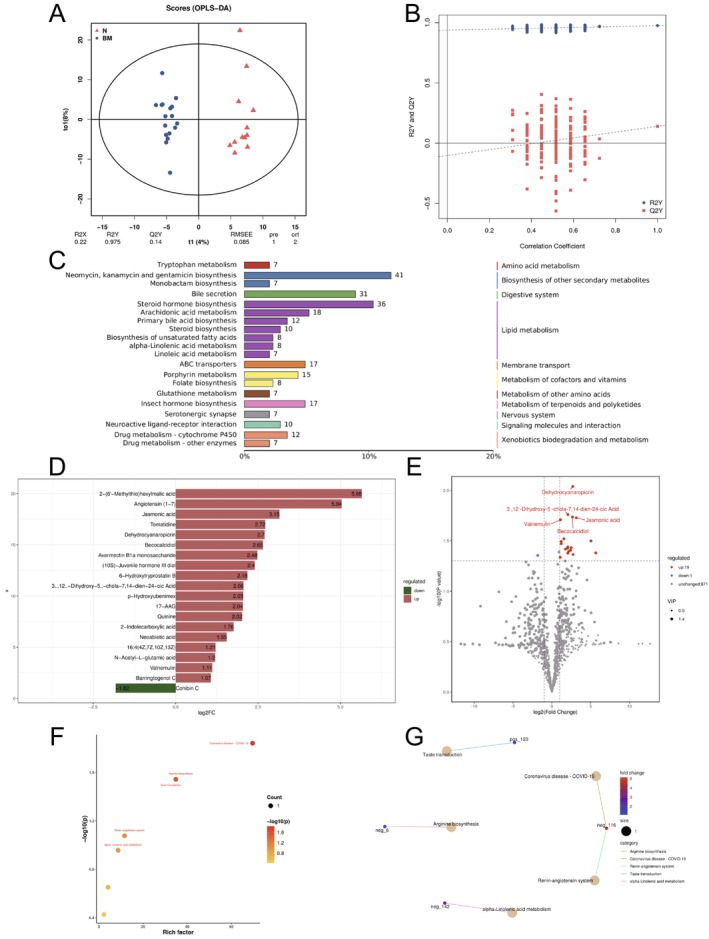
Non‐targeted metabolomics analysis of fecal samples from NSCLC patients. (A, B) OPLS‐DA showing the separation of metabolic profiles between non‐metastatic (*N*) and brain metastatic (BM) NSCLC patients. (C) Classification of metabolites annotated from the KEGG database in the fecal samples of both patient groups. (D) Fold change in the levels of gut microbiota metabolites between the two groups. (E) Volcano plot illustrating the enrichment of differential metabolites, with red points indicating significantly upregulated metabolites in brain metastasis patients. (F, G) KEGG pathway analysis and enrichment network of differential metabolites.

To further elucidate the role of gut microbiota and its metabolites in the brain metastasis of NSCLC, we conducted a non‐targeted metabolomics analysis to profile the metabolic landscape of the gut microbiota. Excluding one sample from the brain metastasis group due to quality control failure, we performed both qualitative and quantitative metabolomic analyzes on 29 samples. Using the LC‐QTOF platform, we annotated a total of 991 metabolites. OPLS‐DA was employed to compare the primary distribution patterns of metabolites between the two groups, revealing significant differences in the metabolic profiles of the gut microbiota (Figure 2A,B). These metabolites were annotated using databases such as KEGG, HMDB, and LIPID MAPS, which suggested that lipid metabolism was the most significantly enriched pathway (Figures 2C and S2).

In the feces of brain metastasis patients, the levels of 19 metabolites, including angiotensin (1–7), jasmonic acid, tomatidine, dehydrocyanaropicrin, and becocalcidiol, were significantly upregulated. In contrast, Cohibin C was upregulated in non‐metastasis patients, suggesting that cohibin C may have a protective role in brain metastasis (Figure 2D,E). To further interpret these findings in terms of biological phenotypes, we performed enrichment analysis on the KEGG annotations of the differential metabolites. The resulting enrichment bar chart showed that these metabolites were involved in various pathways, including Coronavirus disease—COVID‐19, Taste transduction, Arginine biosynthesis, the Renin‐angiotensin system, and alpha‐Linolenic acid metabolism (Figure 2F,G). These findings suggest that the metabolic alterations in the gut microbiota play a significant role in the progression of NSCLC brain metastasis, with specific metabolites and metabolic pathways potentially serving as biomarkers for the disease.

### 3.4 Multi‐Omics Analysis Reveals the Correlation of Gut Microbiota and Metabolites in NSCLC Brain Metastasis

**FIGURE 3:**
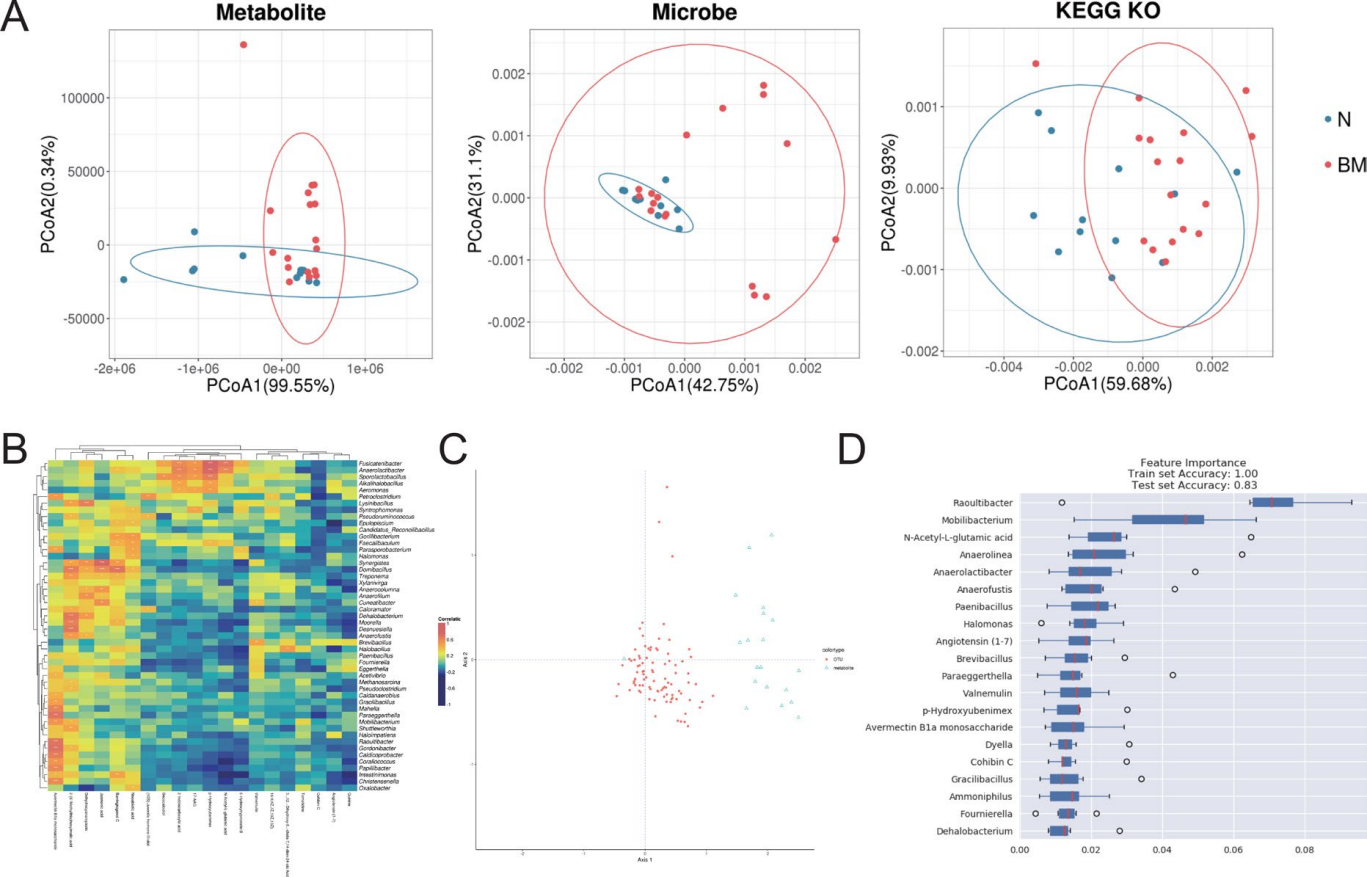
Correlation of gut microbiota and metabolites in NSCLC brain metastasis. (A) Principal Co‐ordinates Analysis (PCoA) showing the differences in metabolite, microbe, and function. (B) Heatmap illustrating the correlation between the abundance of differential metabolites and species. Blue represents negative correlations, while red indicates positive correlations. The color intensity reflects the strength of the correlation (*p < 0.05, **p < 0.01, ***p < 0.001). (C) Coinertia analysis displaying the relationships between differential metabolites and microbial species. (D) Feature importance ranking of key biomarkers in distinguishing non‐metastatic and brain metastatic NSCLC patients using the random forest model.

To explore the associations between gut microbiota and metabolites, we performed an integrative analysis of the metagenomics and metabolomics data. Dimensionality reduction and comparative analysis of multi‐omics data revealed significant inter‐group differences in gut microbiota, metabolites, and gene functions between the two patient groups. This suggested that the occurrence of NSCLC brain metastasis may involve specific biological mechanisms (Figure 3A).

The results also showed significant correlations between gut microbiota metabolites and species abundance (Figure 3B). For example, the SCFA‐producing bacterium *Fusicatenibacter* was strongly correlated with the levels of *N*‐acetyl‐L‐glutamic acid, *p*‐hydroxyubenimex, and 2‐indolecarboxylic acid (*p* < 0.001). Additionally, there was a significant negative correlation between the metabolite angiotensin (1–7) and *Epulopiscium*, a bacterium previously reported to be associated with malignant pleural effusion [18]. Through coinertia analysis, we jointly analyzed differential species and metabolites to reveal their interrelationships and the distribution of different microbial taxa (Figure 3C). The results demonstrated a close association between the gut microbiota and metabolites, with a concentrated distribution of microbial species suggesting ecological niche overlap. The widespread distribution of metabolites might indicate that metabolite production is influenced by a diverse microbial community.

We further applied machine learning techniques, specifically a Random Forest model, to integrate metabolomics and metagenomics data. Feature importance ranking was performed to identify key biomarkers predictive of brain metastasis. The feature importance analysis revealed that both microbial species (e.g., *Raoultibacter*, *Mobilibacterium*) and metabolites (e.g., *N*‐acetyl‐L‐glutamic acid) play critical roles in the prediction of brain metastasis (Figure 3D), suggesting a complex interplay between the microbiome and metabolic pathways. These findings provided a deeper understanding of the molecular signatures associated with brain metastasis and underscore the potential of integrating multi‐omics data for biomarker discovery.

## 4 Discussion

Brain metastasis is one of the most common and fatal complications of NSCLC, but its underlying mechanisms remain poorly understood. This study provides novel insights into the potential role of gut microbiota and its metabolites in the development of brain metastasis in NSCLC through multi‐omics assays.

Our results demonstrated that the gut microbiota of NSCLC patients with brain metastasis significantly differs from that of patients without distant metastasis. Specifically, we observed higher alpha diversity indices, including Chao1 and ACE, in patients with brain metastasis, suggesting a more complex and diverse microbial composition in the gut of these patients. This is consistent with previous studies indicating that microbiota diversity may correlate with tumor progression and metastasis in various cancer types, including lung cancer [19, 20]. In addition, beta diversity analysis revealed significant differences in the overall microbial community structure between the two groups. This dysbiosis may create an environment conducive to tumor progression and metastasis by modulating immune responses, influencing the tumor microenvironment, or altering systemic metabolic pathways [21].

Notably, we identified specific bacterial genera whose abundance was significantly altered in brain metastasis patients. For example, genera such as *Paenibacillus*, *Fournierella*, and *Adlercreutzia* were significantly enriched in the gut of brain metastasis patients. These bacteria have been previously implicated in immune regulation and inflammation, both of which are critical processes in cancer progression and metastasis [22]. Interestingly, our metagenomic analysis revealed that *Fusobacteria* was enriched in the non‐metastasis group. However, similar studies in colorectal cancer have shown a significant increase in the abundance of *Fusobacterium*, which may promote tumorigenesis by inducing DNA damage and altering the immune environment [23]. In breast cancer, *Fusobacterium* has also been associated with poor prognosis and potential metastasis [24]. These similarities suggest that this subspecies may play a broader and more diverse role in cancer metastasis. The overrepresentation of such bacteria suggests that they may play a role in promoting metastasis through immune modulation or the production of metabolites that facilitate tumor cell migration and survival [25, 26]. Conversely, some beneficial microbial species, known for their anti‐inflammatory properties, were diminished in the gut microbiota of patients with brain metastasis, further supporting the hypothesis that gut dysbiosis contributes to the metastatic process.

The functional capacity of the gut microbiota, as assessed through metabolic profiling, revealed significant differences in the levels of key metabolites between the two groups. Notably, SCFAs, such as acetate, butyrate, and propionate, which are produced by gut microbiota, were found to be significantly associated with microbial taxa involved in immune regulation. SCFAs have been shown to influence immune responses by modulating the differentiation and function of T cells and macrophages [27, 28]. Similar studies have also shown that the total concentration of SCFAs in the stool is significantly lower in patients with brain metastasis compared to patients with NSCLC at the early stage and healthy controls [19]. In the context of cancer, these metabolites have been linked to both immune suppression and tumor progression, depending on their concentrations and the types of immune cells they interact with [29, 30]. In our study, the metabolic profiles in brain metastasis patients revealed an altered balance of these metabolites, suggesting a potential link between microbial metabolites and brain metastasis development.

Further analysis demonstrated a strong correlation between gut microbiota and its metabolites, reinforcing the notion that the gut microbiome and its metabolic products influence not only local tumor growth but also distant metastasis. Current studies have shown that the gut microbiota and its metabolites significantly influence various aspects of cancer metastasis. It has been recognized that gut microbiota may also impact metastasis by regulating key factors such as immune cell recruitment, endothelial permeability, and tumor cell migration. For example, studies have demonstrated that the gut microbiota can influence the metastatic spread of colon cancer to the liver through modulation of the immune system [31]. Additionally, metabolites like bile acids and tryptophan derivatives can alter immune cell function, modulate epithelial barrier integrity, and influence tumor progression [32, 33].

It is worth noting that similar studies have also found that the presence of certain microbial species and their metabolites may promote or inhibit the spread of cancer cells to the brain. For instance, *Fusobacterium nucleatum* , a bacterium commonly found in the gastrointestinal tract, has been shown to facilitate the migration of cancer cells and promote metastatic spread by modulating immune responses and increasing the production of inflammatory cytokines [24]. Additionally, metabolites such as SCFAs have been reported to enhance blood–brain barrier permeability, thereby facilitating the entry of circulating tumor cells into the brain [34]. Our findings align with emerging research indicating that metabolites produced by the gut microbiota can impact the permeability of the blood–brain barrier, thus facilitating the entry of metastatic cells into the brain [35, 36]. The observed upregulation of metabolites such as angiotensin (1–7) in the feces of brain metastasis patients could also play a role in modulating vascular permeability and promoting the survival of tumor cells in the brain [37].

The integrative multi‐omics analysis, combining metagenomics and non‐targeted metabolomics, provided a comprehensive view of the complex interactions between gut microbiota and its metabolites in brain metastasis. By utilizing advanced machine learning models, we identified several biomarkers, including *Raoultibacter*, *Mobilibacterium*, and *N*‐acetyl‐L‐glutamic acid, which have the potential to predict brain metastasis in NSCLC patients. This approach underscores the promise of microbiota‐based biomarkers in clinical applications, particularly for early detection and monitoring of brain metastasis in NSCLC patients [38, 39]. However, several limitations of our study should be acknowledged. First, as a single‐center study with a relatively small sample size, our findings may be subject to selection bias, and the results require validation in larger, multi‐center cohorts to enhance their generalizability and applicability. Additionally, although we identified specific gut microbiota and metabolites associated with brain metastasis in NSCLC, we did not conduct biological experiments to validate these associations. Further large‐scale studies are needed to confirm these findings and explore the underlying mechanisms in more detail.

## 5 Conclusions

This study highlights the significant role of gut microbiota and its metabolic products in the development of brain metastasis in NSCLC patients. We identified key microbial species and metabolites associated with brain metastasis, providing evidence that gut microbiota dysbiosis may contribute to the metastatic spread of NSCLC. Our findings also suggest that the gut microbiota and its metabolites could serve as potential biomarkers for predicting and monitoring brain metastasis in NSCLC. These results offer valuable insights into the gut‐brain‐tumor axis and open up new possibilities for microbiota‐based therapeutic strategies to prevent or treat brain metastasis. Future research should aim to validate these findings in larger cohorts and explore the potential of microbiota modulation as a therapeutic approach to managing NSCLC brain metastasis.

## Author Contributions

**Chen‐Guang Liu, Mei‐Xi Lin, Yu Xin, Man Sun:** data curation, analysis, interpretation, investigation, writing – original draft. **Jia Cui, Dan Liu, Dan Zang, Jun Chen:** conceptualization, funding acquisition, methodology, data curation, and writing – review and editing.

## Conflicts of Interest

The authors declare no conflicts of interest.

## Supporting information


**Data S1.** Supporting Information.

## Data Availability

The data that support the findings of this study are available from the corresponding author upon reasonable request.
