# Supplementary material for: Metagenomics and Non‐Targeted Metabolomics Reveal the Role of Gut Microbiota and Its Metabolites in Brain Metastasis of Non‐Small Cell Lung Cancer
Source: Thorac Cancer. 2025 Apr 22;16(8):e70068. doi: 10.1111/1759-7714.70068 (PMC12014518; doi:10.1111/1759-7714.70068)
Supplement: Supplementary file 1 — Data S1. Supporting Information. [file TCA-16-e70068-s001.docx]

Supplementary Figures


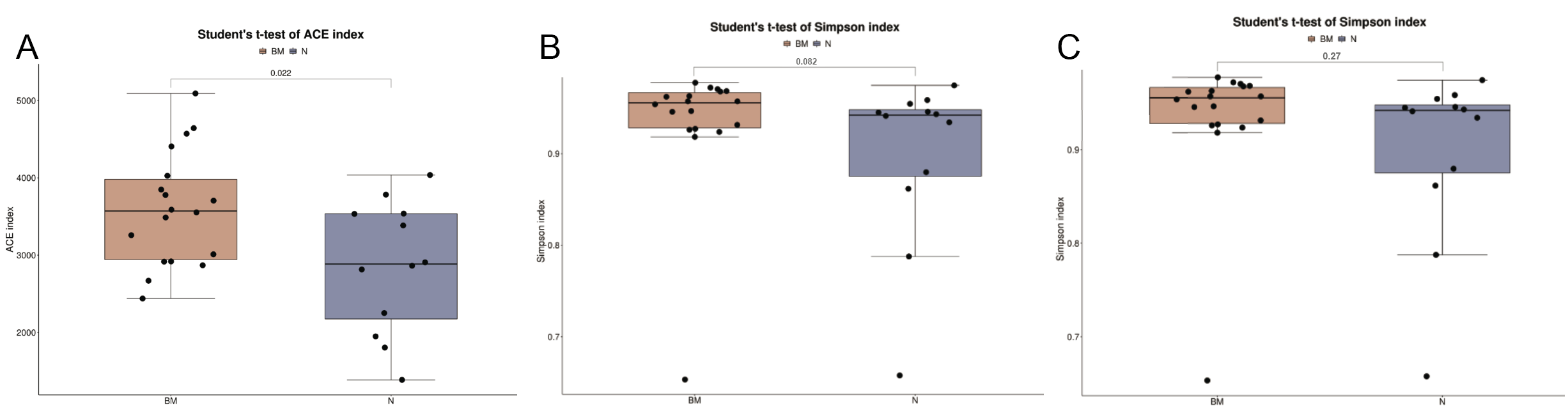


Figure S1. The differences in ACE index (A), Shannon index (B) and Simpson index (C) between NSCLC patients without distant metastasis (N) and those with brain metastasis (BM) were based on α-diversity analysis.


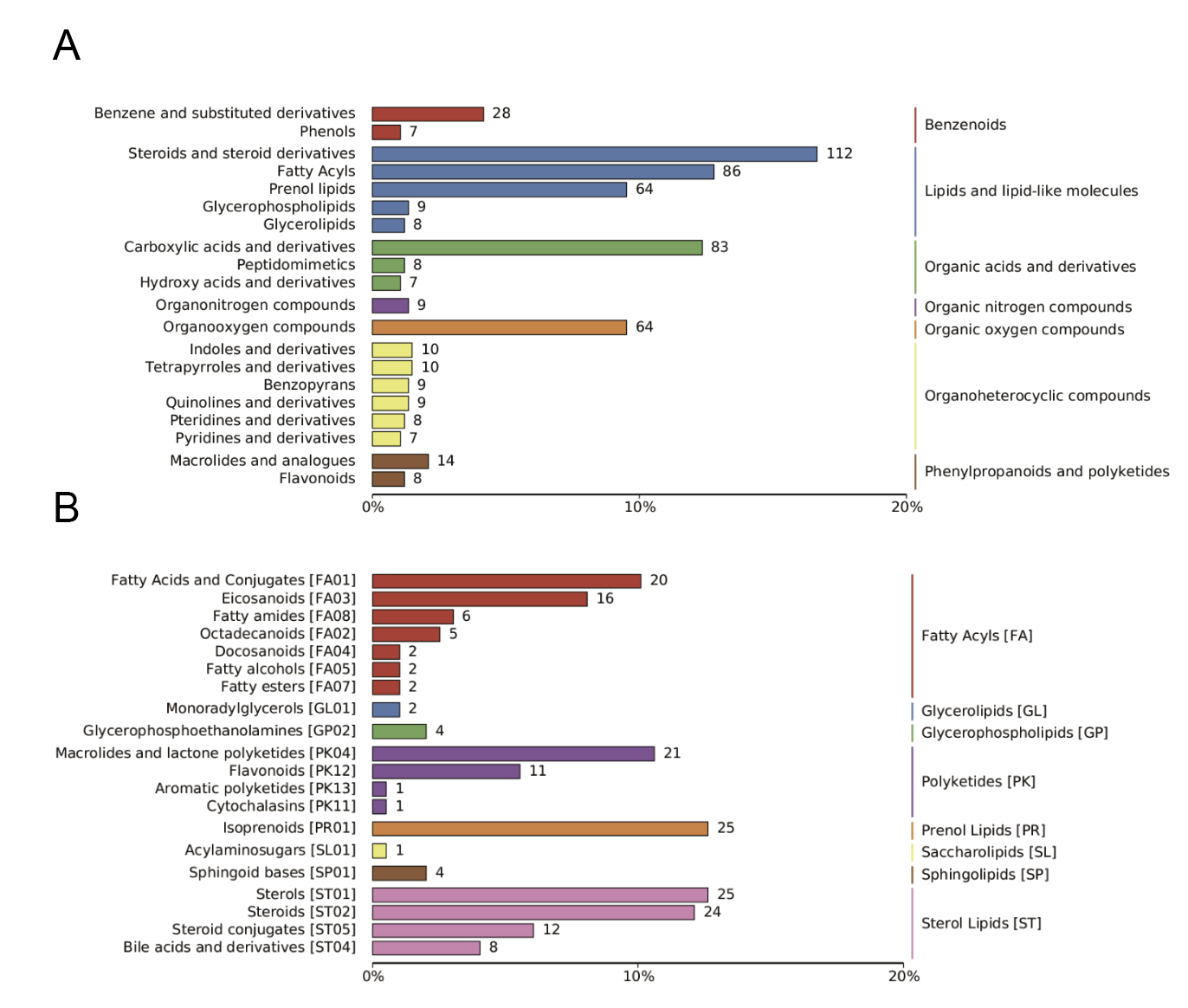


Figure S2. Metabolite classification information annotated by HMDB (A) and LIPID MAPS (B) database in stool samples of NSCLC patients.
